# The Perfection of Human Happiness

**Published:** 1890-10-25

**Authors:** 


### The Doctor's Pulpit.

### THE PERFECTION OF HUMAN HAPPINESS.

Our view of the perfection of human happiness matters little, but its attainment by the majority of people matters much. If life is not to live but to be well, then he who points the way to health indicates the path of perfection also. Thus a considerate regard for one's own powers, coupled with moderation in matters of diet, bodily health and all that promote it, to wit, sufficient exercise, and the avoidance of unnecessary drawbacks produced by excess of any kind, being sound health principles their enunciation is in effect an indication of the road to the perfection of human happiness. Health alone will not, however suffice. We have, therefore, to remember and to act upon the knowledge that there is nothing better for a man than that he should make his soul enjoy good in his labour. All good things around us are sent by Heaven, and there is no better gift to man than the fullest powers to undertake earnest labour, and to adequately persist in it so long as life continues. If the majority of people could only believe and act upon this principle how much happier the world would be. Dyspepsia, irritability, moroseness, uncharitableness, and a host of other failings would become merely a name, because they would then almost, if not quite, disappear from the daily life of mankind.

But, apart from eating and drinking, Solomon wisely lays it down that a man should make his soul enjoy good in his labour. Good work is usually work into which the worker puts his heart and soul. No doubt many people have to earn their living by occupations which are not altogether congenial; still. the mere fact that they are not congenial should make the worker more strenuous in their discharge. The majority of people, however, do not suffer from this drawback, and the more a man puts his heart into his work the better will that work be, and the larger will be the results he is able individually to secure. Solomon had an opportunity which probably no other man has possessed of trying everything in the way of amusement, and he says, and says truly, that, after all, the most satisfying form of enjoyment is that where a man makes his soul enjoy good in his labour. Of course, the whole of the day of most men is not given entirely to work of a remunerative character, and it is therefore well—we are inclined to think it is also necessary—that every thinking man and woman should mark out for themselves some kind of work, if possible for the good of others, to fill a portion of their leisure hours. Everywhere some kind of this form of work may be met with, and it is to the advantage of the individual that the choice is so wide that each man may find satisfaction by selecting that which suits him best. Work is honourable, work is ennobling, work is inspiriting, work makes a man feel that he is able to speak with his enemy in the gate, and to hold his head high when he converses with his fellows. Work itself is the noblest form in which man can show his gratitude to his Creator, because it enables him to labour, with whole-hearted devotedness, to leave the world a little better than he, however, found it.

No man can make his soul enjoy good in his labour if he suffers from the effects of overwork. The amount of work that can be performed safely by each individual cannot be generally determined, for each has a definite power of doing work, and what one man accomplishes easily may prove positively injurious to his fellow. The time of life also makes a difference, and it is manifest that a young man of twenty-five will often do with ease that which must prove highly detrimental to the same man after forty-five. It is a wise provision of nature that each person speedily learns when he is doing too much. To leave off with a feeling of lassitude or irritability, and a growing sense of a gradual loss of power, with increased difficulty in applying the mind to the work in hand; headache accompanied or followed by sleeplessness; all these are danger signals thrown out by nature, whose warnings must never be neglected. The man or woman so warned who still endeavours to accomplish the same amount of work will surely break down, the strain making itself felt, sooner or later, on the weakest organ, whether it be heart, or brain, or kidneys. The heart is usually the first to exhibit signs of weakness when the work is of an active kind, one or both of the latter organs being usually affected when great mental strain and worry is involved.

A business man, working hard in an offi e all day, too often lives in a continual state of hurry and excitement. He hurries to different appointments, he hurries to catch the train to and from his office, he hurries home to a dinner party, and so the day is spent. No wonder if the heart and other organs give way under the strain. Nowadays there is too much of a race for wealth at the expense of health, and haste to make money produces far more harm to the individual than any amount of quiet, steady, congenial labour. Persons who allow themselves to be involved in the vortex of the world's whirlpool never realise, and can therefore never enjoy, the perfection of happiness. Men and women who are wise will so arrange their work that no one pursuit is allowed to monopolise more than a reasonable proportion of each day. Variety in work is true recreation, and without it no one can long continue healthy or happy. Not only is a change of work essential, but a proper provision for recreation will be made by those who desire to enjoy the perfection of happiness. There is no sadder sight than that of a person who, having worked hard all his life in one narrow and confined groove, finds when the necessity for work is over that he has no resource left but to continue that work or to suffer an intolerable boredom. Dr. Wilkes advises young people to encourage a hobby. "If you cannot find pleasure in the study of the very many wonders that surround you, if you care not for geology, natural history, or astronomy, collect walking-sticks, buy and cherish old and cracked china, fill up albums and scrap-books, or even gather together autographs and postage-stamps; anything sooner than be idle." Dr. Wilkes is undoubtedly right, for true recreation requires a thorough change of work and also of thought.

Those who have to work in cities should aim at acquiring a taste for gardening and outdoor pursuits. Hence the amateur ornithologist, botanist, etymologist, gardener, and farmer, all find that as soon as work is over they can turn to a pursuit at once healthy, instructive, and full of pleasure. It is quite a mistake for the town resident to get into the habit of spending his vacation in idling at the seaside, or to a greater or less extent to pass his holidays without any definite pursuit of any kind. Travelling is useful and beneficial, and should not be neglected; but those who are wise will take care to secure a cottage in the country, so that they may rejuvenate their energies and recruit their strength by securing not only a change of scene, but the most perfect of all recreations afforded by the sense of complete and independent leisure, full of enjoyment because there is enough to be done to make each day pass rapidly and pleasantly. The man who successfully attains this will make his soul enjoy good in his labour. Having done this, he will in the highest and best sense have secured the perfection of human happiness.

